# Microarray analysis identification of key pathways and interaction network of differential gene expressions during osteogenic differentiation

**DOI:** 10.1186/s40246-020-00293-1

**Published:** 2020-11-25

**Authors:** Fatemeh Khodabandehloo, Sara Taleahmad, Reza Aflatoonian, Farzad Rajaei, Zahra Zandieh, Marjan Nassiri-Asl, Mohamadreza Baghaban Eslaminejad

**Affiliations:** 1 Department of Molecular Medicine, Qazvin University of Medical Sciences, Qazvin, Iran; 2 Department of Stem Cells and Developmental Biology, Cell Science Research Center, Royan Institute for Stem Cell Biology and Technology, ACECR, Tehran, Iran; 3 Department of Endocrinology and Female Infertility, Reproductive Biomedicine Research Center, Royan Institute for Reproductive Biomedicine, ACECR, Tehran, Iran; 4 Cellular and Molecular Research Center, Research Institute for Prevention of Non-Communicable Disease, Qazvin University of Medical Sciences, Qazvin, Iran; 5 Department of Anatomy, School of Medicine, Iran University of Medical Sciences, Tehran, Iran

**Keywords:** Bone mesenchymal stem cells, Bioinformatics analysis, Osteogenic differentiation, Protein-protein network

## Abstract

**Background:**

Adult bone marrow-derived mesenchymal stem cells (BM-MSCs) are multipotent stem cells that can differentiate into three lineages. They are suitable sources for cell-based therapy and regenerative medicine applications. This study aims to evaluate the hub genes and key pathways of differentially expressed genes (DEGs) related to osteogenesis by bioinformatics analysis in three different days. The DEGs were derived from the three different days compared with day 0.

**Results:**

Gene expression profiles of GSE37558 were obtained from the Gene Expression Omnibus (GEO) database. A total of 4076 DEGs were acquired on days 8, 12, and 25. Gene ontology (GO) enrichment analysis showed that the non-canonical Wnt signaling pathway and lipopolysaccharide (LPS)-mediated signaling pathway were commonly upregulated DEGs for all 3 days. KEGG pathway analysis indicated that the PI3K-Akt and focal adhesion were also commonly upregulated DEGs for all 3 days. Ten hub genes were identified by CytoHubba on days 8, 12, and 25. Then, we focused on the association of these hub genes with the Wnt pathways that had been enriched from the protein-protein interaction (PPI) by the Cytoscape plugin MCODE.

**Conclusions:**

These findings suggested further insights into the roles of the PI3K/AKT and Wnt pathways and their association with osteogenesis. In addition, the stem cell microenvironment via growth factors, extracellular matrix (ECM), IGF1, IGF2, LPS, and Wnt most likely affect osteogenesis by PI3K/AKT.

## Introduction

Mesenchymal stem cells (MSCs) are multipotent and nonhematopoietic stromal cells that have the ability for self-renewal [1]. MSCs are isolated from different sources and their characteristics depend on the source [2, 3] from which they are obtained. It is necessary to identify the molecular mechanisms for osteogenic differentiation in bone marrow MSCs (BM-MSCs) [4] that have a high potential for osteogenesis. Because of their ability to differentiate into osteoblasts, these cells have been extensively used for regenerative medicine and the cure of bone disorders. Osteogenic differentiation is a programmed process, and the most current knowledge has been obtained by determining the role of genes such as runt-related transcription factors 2 (Runx2), distal-less homeobox 5 (Dlx5), osteocalcin (OCN), and osterix (Osx) [5]. In addition, other factors involved in osteogenic differentiation include bone morphogenetic proteins (BMPs) [6], fibroblast growth factor (FGF) [7], transforming growth factor-β (TGF-β) [6], hedgehog (HH) [8], and microRNAs [9]. However, the role of many genes and the relationship between them during osteogenesis of MSCs is not completely understood, and there is a need to focus on them to improve the knowledge of tissue and bone engineering.

For this reason, several hypotheses have been considered in this study as follows: the first hypothesis is that NF-κB is an important mediator in osteogenesis promotion by toll-like receptor 4 via the BMP2 pathway. Since TLR4 from the lipopolysaccharide (LPS)-mediated pathway plays an important role in osteogenesis. The TLR4 promotes osteogenic differentiation upon stimulation by its ligand LPS [10]. Stimulation of TLR4 by LPS in human dental pulp stem cells (hDPSCs) activated NF-κB by regulating the PI3K/AKT signaling [11]. However, the mechanisms by which NF-κB regulates differentiation of MSCs into osteoblasts are not well known.

The second hypothesis is that the genes in focal adhesion signaling promote osteogenic differentiation, since the extracellular matrix (ECM) can affect osteogenic differentiation via focal adhesion kinase (FAK). Upon binding of the ligand to integrins, FAK becomes activated [12]. Activation of FAK is essential for MSCs to commit to osteoblasts [13]. FAK phosphorylates PI3K and mitogen-activated protein kinases-extracellular signal-regulated kinase-1/2 (MAPK-ERK1/2).

The third hypothesis is that stem-cell niche and microenvironment mediate osteogenesis through the PI3K/AKT signaling pathway, since it seems that the PI3K/AKT is the central pathway in differentiating the MSCs into the osteoblast.

Furthermore, as the role of the Wnt pathway in the differentiation of MSCs into osteoblasts [14, 15], the fourth hypothesis is that both canonical and non-canonical Wnt pathways contribute to the regulation of osteogenesis. Two known signaling pathways exist for Wnt, canonical (Wnt/β-catenin) and non-canonical. The non-canonical pathway contains the Wnt/planar cell polarity and the Wnt/calcium pathways [16, 17]. Therefore, we examined how genes associated with the non-canonical Wnt pathway might influence the differentiation of MSCs into osteoblast. It seems that both the FZD4 and SFRP1 perform their role via both focal and non-focal pathways in osteogenesis.

To test these four hypotheses, we evaluated the gene expression profiling microarray data (GSE37558) at different time points (days 8, 12, and 25) during osteogenesis of MSC and focused on the main biological processes and KEGG pathway enrichment of the differentially expressed genes (DEGs) which were derived from the three different days compared with day 0. The highlighted pathways in our study were including LPS-mediated signaling pathway, focal adhesion, PI3K/AKT, and Wnt pathway which play important regulatory functions during the osteogenesis of hMSCs.

## Results

### Identification of differentially expressed genes (DEGs)

**Fig. 1:**
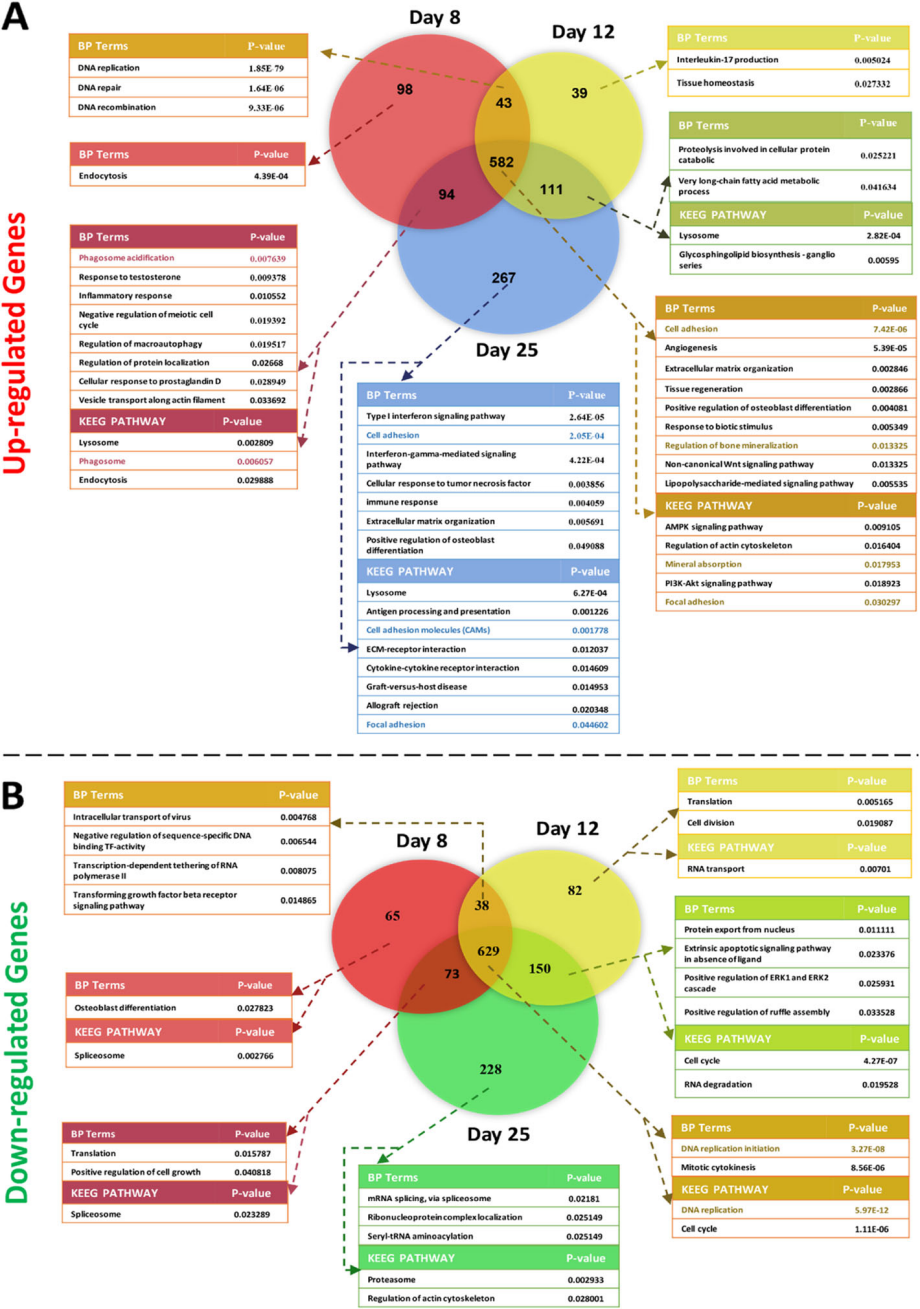
Venn diagram of biological process (BP) and KEGG pathway enrichment analysis on days 8, 12, and 25. **a** Upregulated differentially expressed genes (DEG). **b** Downregulated DEGs. Common biology terms between the biological process and KEGG pathway were shown in the same color

Analysis of microarray data from the GSE37558 study enabled us to identify 4076 total DEGs by GEO2R. There were 1234 upregulated DEGs and 1265 DEGs significantly downregulated based on the criteria of the adjusted *P* values of < 0.01 and |logFC| > 0.5 (Fig. 1a, b). On day 8, there were 817 upregulated DEGs, 775 upregulated DEGs for day 12 and 1054 for day 25. There were 805 downregulated DEGs on day 8, 899 on day 12, and 1080 on day 25. Interestingly, there were 582 DEGs with a maximum similarity that were upregulated and 629 DEGs that had maximum similarity and were downregulated on all 3 days. On day 25, a total of 267 upregulated DEGs showed the most change in expression and 228 downregulated DEGs had the most change in expression (Fig. 1a, b). Therefore, we focused on biological processes (BP) terms and pathways related to these DEGs.

### Gene ontology (GO) term enrichment analysis

GO biological processes (BP) analysis showed that angiogenesis, the non-canonical Wnt signaling pathway, and lipopolysaccharide (LPS)-mediated signaling pathway were commonly upregulated DEGs on days 8, 12, and 25 (Fig. 1a). DNA replication initiation and mitotic cytokinesis were the most common downregulated DEGs for all 3 days (Fig. 1b). The GO terms including molecular function (MF) and cellular component (CC) are also shown in Supplementary Fig 1.

### KEGG pathway analysis

According to KEGG pathway analysis, the PI3K-Akt signaling pathway, and focal adhesion were the most commonly upregulated DEGs for days 8, 12, and 25 (Fig. 1a) DNA replication and the cell cycle were the most commonly downregulated DEGs for all 3 days (Fig. 1b). Focal adhesion was also upregulated on day 25 (Fig. 1a).

### Construction of the protein-protein interaction (PPI) network and screening of modules

**Fig. 2:**
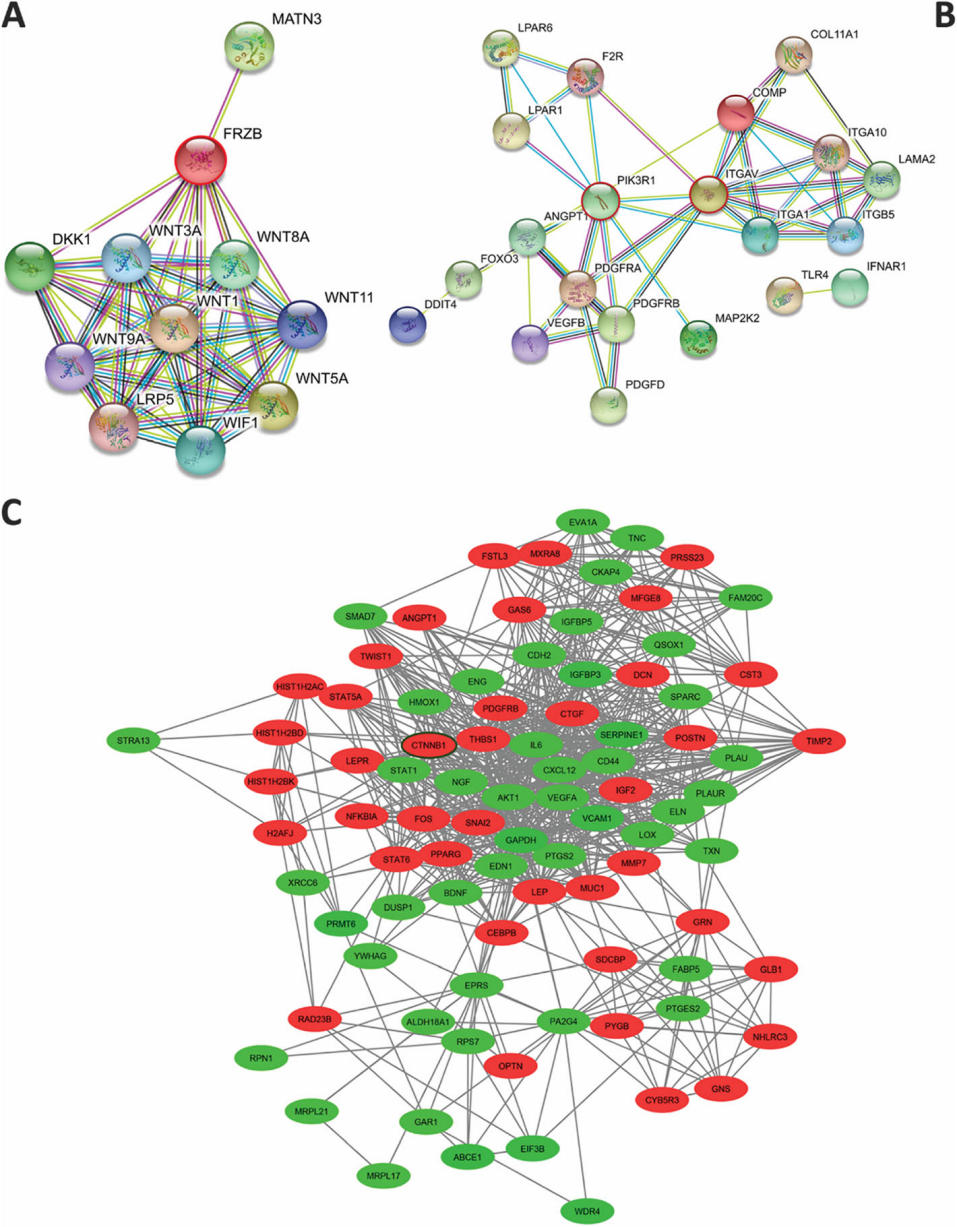
Protein-protein interaction (PPI) network (STRING). **a** FRZB. **b** 14 genes involved in the PI3K/AKT pathway. The red nodes indicated upregulation DEGs, and the green nodes indicated downregulation DEGs. **c** Presence of β-catenin in the protein-protein interaction (PPI) network. Module 4 (day 8).

We used Cytoscape software to visualize the PPI network of the DEGs for the testing days (days 8, 12, and 25). The modules were extracted using MCODE according to the number of nodes that were > 4 and a node score of > 4. Enrichment analyses of the BPs and the KEGG pathway of modules were performed (Supplementary Table 1). Supplementary Table 1 list the modules of DEGs related to osteogenesis from BP and KEGG pathway for days 8, 12, and 25. Of note, the cell cycle and MAPK were enriched for all of the assessed days, whereas the Wnt pathway was enriched only on days 8 and 25. Supplementary Table 2 provides a list of the top 10 hub proteins identified by CytoHubba from Cytoscape for each of the days. The association of the hub genes with the Wnt pathway was investigated. We focused on the CTNNB1(β-catenin) as the hub protein involved in the Wnt signaling in upregulated DEGs for days 8 and 25. CTNNB1 was shown in module 4, day 8 (Fig. 2c), and module 4, day 25 (Supplementary Fig 2).

### Validation of microarray data

**Fig. 3:**
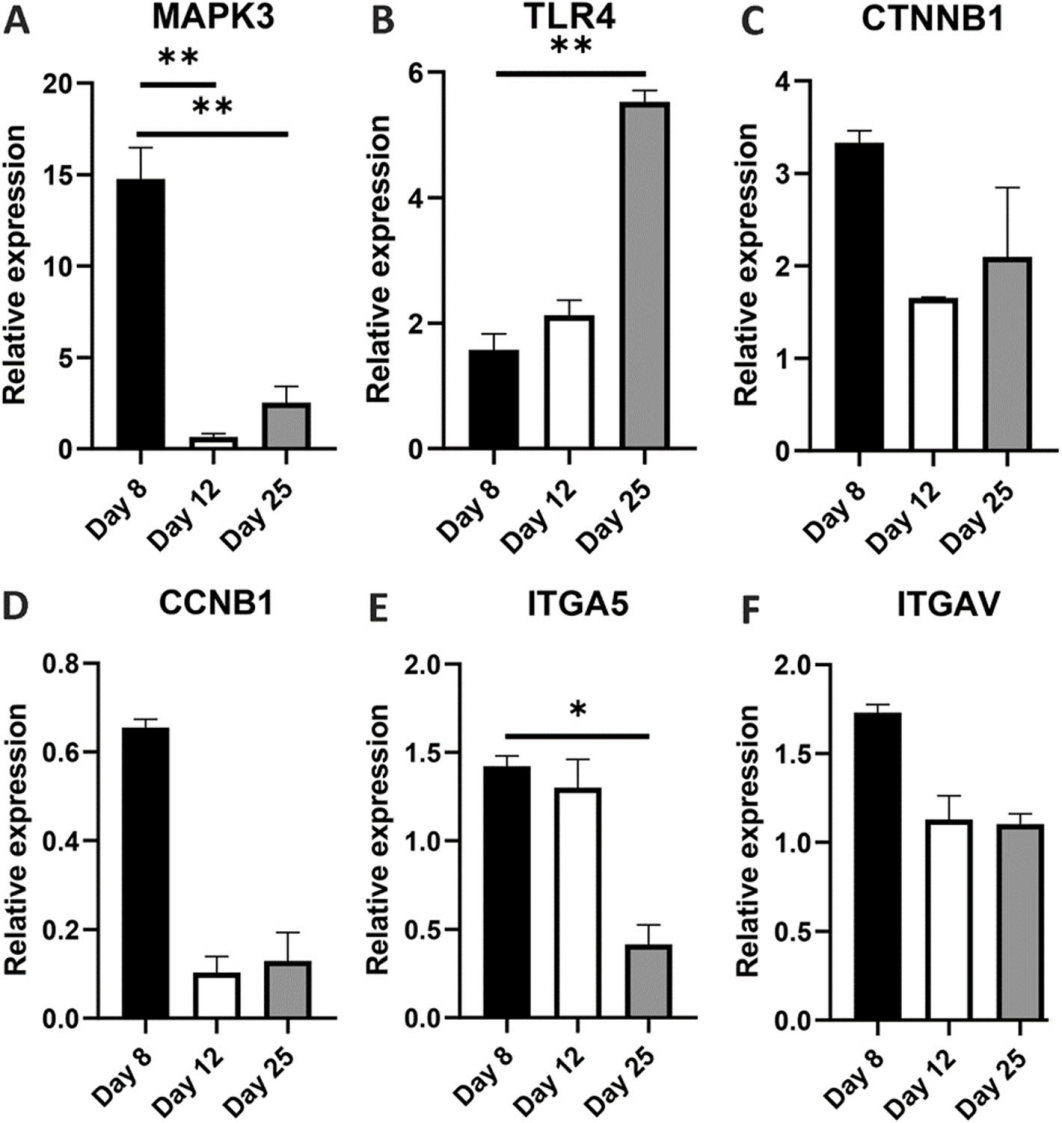
Real-time PCR validation of candidate mRNA at days 8, 12, and 25. **a–f** the mRNA levels of MAPK3, TLR4, CTNNB1, CCNB1, ITGA5, and ITGAV were detected by real-time PCR (*n* = 3). All data are presented as mean ± SEM. **P < 0.01

For verification of microarrays data by real-time PCR, we selected some candidate transcripts for real-time PCR at days 8, 12, and 25. MAPK3 expression was higher in differentiated MSCs at day 8 compared to days 12 and 25 (Fig. 3a). TLR4 expression on day 25 remarkably was enhanced rather than day 8 (Fig. 3b). The CTNNB1 expression at 3 days increased compared to the control group but there were no significant differences between the three groups (Fig. 3c). Time-dependent changes in the expression of the MAPK3, TLR4, CCNB1, and ITGA5 genes were concordant with array results (Fig. 3a, b, d, e), and upregulation of CTNNB1 and ITGAV genes also were in agreement at 3 days with our results (Fig. 3c, f).

## Discussion

Osteogenic differentiation is a complex process where the interaction between genes and pathways has not been fully discovered. According to the International Society of Cell Therapy protocol, MSCs should possess the following three characteristics: (i) have the capability to adhere to plastic surfaces; (ii) test positive for CD90, CD73, and CD105 and negative for CD79, CD19, CD45, CD34, CD14 or CD11b, and HLA-DR; and (iii) have the potential to differentiate into osteoblasts, chondrocytes, and adipocytes in vitro [18]. In this study, we intended to gain further insight into the identification of hub genes and key pathways during the early, middle, and late stages of osteogenic differentiation (days 0, 8, 12, and 25) as common and individual pathways.

### Both canonical and non-canonical Wnt pathways contribute to regulation of osteogenesis

The Wnt pathway plays a role in the migration, growth, cell fate determination, differentiation, and [19, 20] bone differentiation [21]. There are four upregulated genes (*FRZB*, *FZD4*, *SFRP*, and *FZD1*) in the non-canonical Wnt pathway. This pathway is frequently upregulated on days 8, 12, and 25 (Fig. 1a). *FRZB* had the highest logFC for 3 days. The overexpression of *FRZB* by the Wnt/CaMKII pathway promoted osteogenic but not by activation of the canonical pathway [22]. FRZB appears to be important in the gene network (Fig. 2a). Its interaction with canonical and non-canonical ligands has been reported. FZD4 belongs to the Frizzled (FZD) family. Binding of Wnt to FZD4 activates canonical Wnt/β-catenin signaling and promotes osteogenic differentiation. Recent studies have shown that miR-139-5p binds to CTNNB1 and FZD4, it reduces their expression and then osteogenic differentiation is decreased [23]. It was reported that mechanical stimulation promoted osteogenesis by the Wnt5a/FZD4 pathway in BM-MSCs via the non-canonical Wnt pathway [24]. SFRP1 is another upregulated gene in this pathway that has a role in the inhibition of both the canonical and non-canonical pathways [25].

### NF-κB is an important mediator in osteogenesis promotion by toll-like receptor 4 via the BMP2 pathway

**Fig. 4:**
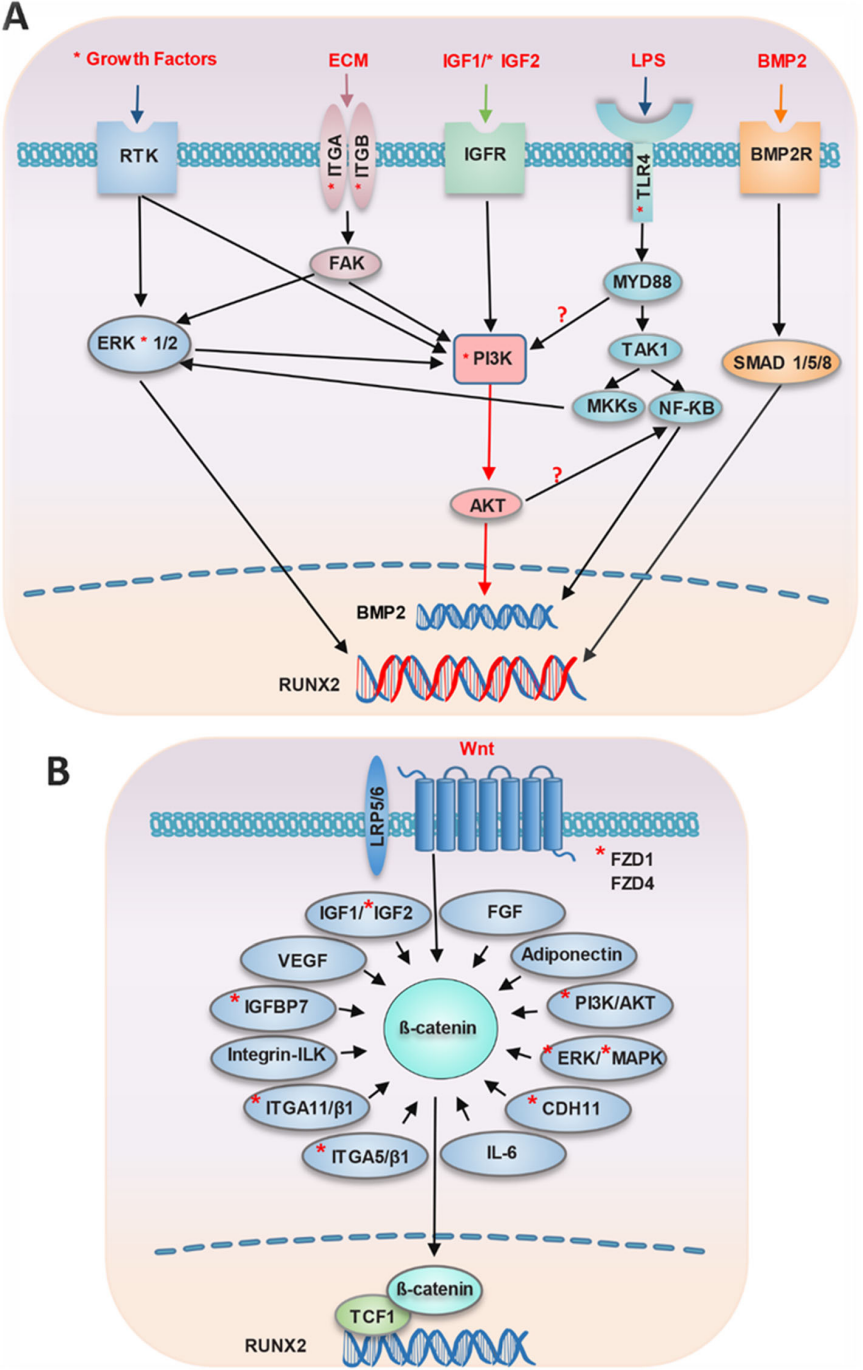
Model of PI3K/AKT regulation and Wnt/β-catenin in osteogenic differentiation. Important pathways in osteoblast that promote osteogenesis via PI3K/AKT and β-catenin. **a** PI3K/AKT and its relationship with growth factors, ECM attachment, IGF1, IGF2, LPS, and BMP2 are illustrated in the top portion of this figure. The question mark next to PI3K/AKT/NF-κB indicates whether PI3K/AKT plays a significant role during osteogenesis directly via BMP2 signaling or indirectly through the upregulation of NF-κB. **b** The interaction or connections of genes and pathways with β-catenin are shown. Wnt/β-catenin interacts or is affected by FGF, IGF-1/IGF-2, IGFBP7, VEGF, integrin-ILK, ITGA11/β1, ITGA5/β1, CDH11, ERK/MAPK, PI3K/AKT, IL-6, and adiponectin. The red star represents commonly upregulated genes on all 3 days. Only expression of MAPK3 (ERK1) at day 8, ITGA5 on days 8 and 25, and both IGFBP7 and ITGA11 at day 25 are represented. ECM, extracellular matrix; PI3K, phosphoinositide 3-kinase; MKK, MAP kinase kinases

LPS-mediated signaling pathway was another BP that was upregulated for all 3 days (Fig. 1a). There were five genes involved in this BP term (*IL18*, *NFKBIA*, *TLR4*, *SCARB1*, and *CD14*). TLR4 and CD14 from this pathway are two genes that are involved in the TLR4 signaling pathway. Three accessory proteins, including MD2, LBP, and CD14, have roles in TLR4 activation. LBP and CD14 facilitate the transfer of LPS to the TLR4/MD2 complex [26]. A study reported that TLR2 and TLR4 ligands (peptidoglycan and LPS, respectively) and TNF-α increase osteogenic differentiation via activation of NF-κB in human adipose tissue-derived stem cells (hADSC) [27]. Taken together, these results suggest that NF-κB is an important transcription factor in the regulation of osteogenesis (Fig. 4a). Hess et al. have demonstrated that NF-κB activation which is induced by TNF-α promotes osteogenic differentiation by increasing BMP-2 and alkaline phosphatase (ALP) expression [28].

### Stem-cell niche and microenvironment mediate osteogenesis through the PI3K/AKT signaling pathway

We observed that the PI3K/AKT pathway was upregulated in every 3 days. There were 21 genes involved in this pathway (Fig. 2b). Recently, results of a study have shown that platelet-derived growth factor (PDGF) increased osteogenic differentiation induced by TGF-β. However, PDGF alone did not affect osteogenic differentiation; rather, there was synergic cross-talk between the PI3K/AKT (PDGF mediated( and the mitogen-activated protein kinase (MAPK)/ERK kinase (TGF-β mediated( pathways [29]. It was reported that the suppressor of PDGF promoted adipogenesis via PI3K signaling [30]. We observed that, following stimulation of growth factors *PDGFD* and *VEGFB*, the receptor tyrosine kinases PDGFRA and PDGFRB were activated, followed by upregulation of mitogen-activated protein kinase 2 (MAP2K2) of MAPK (for more details, see the next section) and phosphoinositide-3-kinase regulatory subunit 1 (PIK3R1) that belongs to the PI3K pathway (Fig. 4a).

Although the PI3K/AKT signaling pathway is involved in the osteogenesis process in humans and mice [31, 32]. In mice, the role of this pathway is shown in endochondral ossification [33], this pathway also has a role in many MSC functions [34]. In vitro, AKT contributes to chondrogenesis and osteoblast development in metatarsal growth isolated from mice [35]. It has been reported that the activation of PI3K/AKT signaling was important in noncaveolar cholesterol-rich membrane raft likely for human MSC osteogenesis [31].

Osteogenic differentiation in rat tendon stem cells (TSCs) is affected by prostaglandin E2 that activates PI3K/AKT signaling, resulting in osteogenic differentiation induced by BMP [36]. Interestingly, BMP-2 appears to mediate the effects of the PI3K/AKT pathway on osteogenesis. The role of BMP-2 has been shown in the commitment of progenitors into osteoblasts and in the stimulation of Runx2 and other transcription factors such as Dlx3 and Dlx5 that promote osteogenesis [37, 38]. In another study, it was suggested that AKT might not directly mediate BMP-2 expression; rather, it promotes osteogenesis possibly through a transcription factor such as the NF-κB [36]. Induction of this pathway by insulin-like growth factor (IGF) promoted osteoblast differentiation through BMP. The role of IGFs also in bone formation and development is via the PI3K/AKT pathway [32].

Mukherjee et al. have demonstrated that AKT played a role in all stages of osteogenic differentiation. The results of a study indicated that AKT2, but not AKT1, was important in osteogenic differentiation through BMP-2 that AKT2 stimulated expression of the Runx2 gene [39]. Overall, the PPI results in this pathway have revealed the interaction of the PIK3R1 and integrin subunit alpha V (ITGAV) which are important genes in this network (Fig. 2b).

### Upregulation of involved genes in the MAPK pathway led to osteogenesis regulation

In our study, MAP2K2 from the PI3K-AKT pathway (on day 3) and MAPK3 (on day 8) as a hub gene were upregulated. MAP2Ks, including MEK1 (MAP2K1) and MEK2 (MAP2K2), activate ERK1 (MAPK3) and ERK2 (MAPK1) [40]. Studies have shown that the MAPK pathway is important for bone formation [41–43]. However, the role of MAPKs in osteogenesis is contradictory. A recent study has demonstrated that TRIB3 influenced proliferation and differentiation in the middle stage of differentiation by inhibiting the ERK1/2 [44]. As mentioned above, the molecular mechanism that TLR4 is involved in controlling the fate of MSCs toward osteogenesis is still uncovered. Upon MAP kinase kinases (MKKs) activation in the TLR4 pathway, p38, JNK, and ERK1/2 activated [45]. In the recent study, maximum ERK activation was shown during osteogenesis of hADSCs at day 7 when LPS stimulation was enhanced [46].

It has been recently demonstrated that JNK1 is a negative regulator of osteogenesis through BMP-2 by Runx2 phosphorylation [47]. JNK2 is needed in the late stage of osteogenic differentiation [48]. Interestingly, in one study, the results showed that JNK1 was involved in mineralization in the late stage of osteogenic differentiation and mediated increased expression of IGF2 and VEGFα from proangiogenic factors [49]. p38 is a positive regulator in OCN synthesis [50]. MAPK signaling could be affected by various factors such as growth factors (TGF-β, BMPs, and FGF2), integrins (ECM), and mechanical loading [51], which, in growth factors and integrins, were consistent with our study (Fig. 4a). ERK and p38 MAP kinase could play a role in osteoblast differentiation through phosphorylation of osteogenic differentiation-related genes such as RUNX2, Osx, and DLX5 [51].

### Genes in focal adhesion signaling promote osteogenic differentiation

There were 14 genes (*ITGA1*, *ITGA10*, *ITGB5*, *CTNNB1*, *MYL9*, *VEGFB*, *LAMA2*, *COMP*, *ITGAV*, *PDGFRA*, *PDGFRB*, *PDGFD*, *COL11A1*, and *PIK3R1*) involved in the focal adhesion pathway that upregulated on days 8–25. We have observed the upregulation of integrin subunit alpha 5 (*ITGA5*) on days 8 and 12. Hamidouche et al. noted that FAK/ERK1/2-MAPKs and PI3K signaling pathways promoted osteogenic differentiation through induction of ITGA5 hMSCs [52]. It has been reported that activation of ITGA5 induced both IGF2 and IGFBP2 expressions via FAK, ERK1/2, and PI3K signaling, which resulted in osteogenic differentiation in hMSCs [53]. In another study, cilengitide (a cyclic RGD pentapeptide) is an ITGAV inhibitor [54] that can abolish ossification in BM-MSCs [55]. The interaction between osteopontin and integrin αv/β1 induced osteogenesis and inhibited adipogenesis in MSCs [56]. Therefore, the binding of osteopontin, fibronectin, and other molecules involved in osteogenic differentiation could determine the possible balance between MSCs that commit toward adipogenic or osteogenic differentiation [57]. We have observed the upregulation of cadherin 11 (CDH11) on all 3 days. CDH11 is involved in cell connections and has a role in cell signaling. CDH11 is expressed in osteoblasts osteogenesis. Its role in osteoblast commitment and osteogenic differentiation has been reported [58]. Our data showed that genes related to cell adhesion such as *ITGA11* and insulin-like growth factor binding protein 7 (*IGFBP7*) also upregulated on day 25. Integrin α11 (ITGA11) is a receptor for osteolectin that actives the Wnt pathway and promotes osteogenesis [59]. Recently, Zhang et al. also reported that the *IGFBP7* gene promoted osteogenic differentiation of hBM-MSCs by upregulation of the β-catenin pathway [60].

### Relationship between hub genes and Wnt pathway during osteogenesis

The role of important hub genes including interleukin (IL)-6, AKT1, VEGFA, CDK1, PLK1, CDC20, CCNA2, MAPK3 (on day 8), and CTNNB1 (on days 8 and 25) in the Wnt pathway was studied (Supplementary Table 2).

The inhibitory effects of IL-6 in osteoblast differentiation in rheumatoid arthritis are due to its negative interaction with the Wnt pathway [61]. In a study, Li et al. suggested that the effect of IL-6 on inhibition of osteogenic differentiation is due to its inhibitory effect on the canonical Wnt pathway [62].

AKT1 is an important gene in the PI3K/AKT pathway. Studies have been conducted about the cross-talk between Wnt/β-catenin and PI3K/AKT signaling pathways [63, 64]. Han et al. have reported that inhibition of PI3K/AKT suppressed transcription through β-catenin in glioblastoma cells [64]. β-catenin can be directly phosphorylated at Ser552 by AKT, which separates it from cell-cell contact and increases translocation of β-catenin into the nucleus, both in vitro and in vivo [65].

VEGFA plays a pivotal role in angiogenesis. Numerous studies have assessed the role played by VEGFA in linking osteogenesis and angiogenesis [66, 67]. In osteoblastic and endothelial cells, VEGF induced bone formation through the β-catenin pathway [68]. Inhibition of β-catenin or knockdown of Wnt4 in the MSCs led to the return of proangiogenic effects induced by Wnt signaling [69].

CDK1, PLK1, CDC20, and CCNA2 are genes involved in the cell cycle. Proliferation and differentiation have opposite connections [70]. In agreement with previous studies, the genes related to the cell cycle were downregulated [71, 72]. The association between the Wnt system and genes related to the cell cycle during osteogenesis was less observed. A study suggested that the induction of Wnt/β-catenin, by LRP6 phosphorylation is regulated via Cyclin Y/CDK at the G2/M phase [73]. The canonical Wnt pathway also plays an important role in cell cycle control [74].

MAPK3 is related to the MAPK pathway. The results showed that the ERK interacts with the Wnt/β-catenin signaling pathway. It has also been shown that the ERK pathway is involved in the differentiation of osteoblasts through the regulation of RUNX2, β-catenin, and ATF4 [43]. The Wnt pathway is indirectly impacted by ERK/MAPK signaling via inhibition of GSK-3β by p38, JNK, and ERK [75].

The role of β-catenin was implicated in both canonical Wnt pathways and cell-cell adhesion [16, 76]. In the canonical pathway, frizzled and LRP5/6 are activated by Wnt ligands. In the presence of Wnt ligands, the destruction complex (GSK3, AXIN, and APC) is inhibited and this inhibition helps the stabilization and translocation of β-catenin to the nucleus [77–80]. There is a site on promoter of Runx2 for β-catenin/TCF-1, which activates expression of this gene and promotes osteogenic differentiation [81]. Tornero-Esteban et al. investigated the involvement of the Wnt and possible compensatory mechanisms involve in the osteoarthritis (OA) pathophysiology. They showed the increased levels of β-catenin in OA-MSCs did not accompany increased osteogenic suggesting that compensatory mechanisms are involved in modulating transcriptional of osteogenic differentiation [82]. Due to the important role of β-catenin in the Wnt pathway, in the next section, we evaluated the β-catenin that interacts/or is affected by other pathways.

### The Wnt/β-catenin pathway could interacts/or was affected by other pathways associated with osteogenesis

The interaction between integrin-related signaling molecules and the Wnt pathway suggested that integrin receptors are associated with integrin-linked kinase (ILK) [83]. GSK3β is phosphorylated following the activation of ILK [84]. Activation of Wnt/β-catenin and PI3K-Akt signaling pathways drive osteogenic differentiation upon primed α5β1 integrin using peptides in mesenchymal skeletal cells [85]. Another study showed that osteolectin/α11β1 results in Wnt pathway activation that increased nuclear β-catenin and finally promoted osteogenesis [59]. CDH11 is involved in osteoblast committed into the osteogenic lineage. Interestingly, adipogenesis was not affected by CDH11 and it may be mediated via β-catenin [58]. A relationship between growth factors and the Wnt signaling pathway during osteogenesis has been reported. (IGF)-I and IGF-II can also affect the β-catenin signaling pathway [86, 87]. IGFBP7 also induced osteogenesis at day 25. FGF interacts with the Wnt/β-catenin pathway in osteogenesis during the regulation of the transcription factor of Osx [88]. The role of adiponectin as an adipocytokine has been shown in bone formation through the Wnt/β-catenin pathway [89]. Overall, Wnt/β-catenin interacts or is affected by PI3K/AKT, ERK/MAPK, CDH11, integrins (integrin-ILK, integrin α5β1, integrin α11β1), growth factors (FGF, IGF1/IGF2, IGFBP7, and VEGF), IL6, and adiponectin (Fig. 4b).

## Conclusion

In summary, PI3K and Wnt signaling are important pathways in osteogenic differentiation. IGF2 links integrin, PI3K/AKT, and MAPK (JNK1). The extracellular environment can affect osteogenic differentiation PI3K/AKT mediated via the following: (1) LPS by TLR4; (2) integrins from ECM by the binding of substances such as osteolectin and osteocalcin, and activation of the FAK/MAPK pathway; (3) IGF1 and IGF2 through IGFRs; (4) growth factors via RTK; and (5) beta-catenin via Wnt pathway. Whether PI3K/AKT promotes osteogenesis through BMP2 directly or via NF-κB should be investigated. Taken together, this study provides further insight into the role of signaling pathways and their interaction in determining the fate of mesenchymal stem cells into osteoblast.

## Materials and methods

### Microarray data analysis

Raw data related to expression profiling of GSE37558 [90] were taken from the Gene Expression Omnibus (GEO, http://www.ncbi.nlm.nih.gov/geo/) database. GPL6947 platforms (Illumina HumanHT-12 v3.0 Gene Expression beadchip) were used for the gene expression profiles. In this study, we selected 13 samples from human BM-derived MSCs (hBM-MSCs) that had been cultured in osteogenic differentiation medium at four-time points (days 0, 8, 12, and 25). The control group comprised four replicates from day 0, and the differentiated groups included three replicates for each time point (days 8, 12, and 25). Sample accession (GSM) of GSE37558 related to this study in four different days are shown in Table 1.

**Table 1:** List of samples accession (GSM) used at different four-time points (days 0, 8, 12, and 25)

Time point	Sample accession
0	GSM921574, GSM921575, GSM921576, and GSM921577
8	GSM921581, GSM921582, and GSM921583
12	GSM921584, GSM921585, and GSM921586
25	GSM921587, GSM921588, and GSM921589

### Identification of differential gene expressions (DEGs)

GEO2R (http://www.ncbi.nlm.nih.gov/geo/geo2r/) was used to identify the DEGs between the control group (day 0) and differentiated groups (days 8, 12, and 25). The DEGs were identified with adjusted *P* values of < 0.01 and |logFC| > 0.5.

### Gene ontology (GO) and pathway enrichment analysis

Gene ontology (GO) for biological processes (BP), molecular function (MF), and cellular component (CC) in addition to KEGG pathway enrichment analyses of differential gene expressions were obtained using the Database for Annotation Visualization and Integrated Discovery (DAVID; https://david.ncifcrf.gov) for up- and downregulated DEGs. *P* values of less than 0.05 were considered to be the criteria. DAVID was also used to analyze both GO ontology and the KEGG pathway of the modules. Venn diagrams (http://bioinfogp.cnb.csic.es/tools/venny/index.html) were used to identify up- and downregulated DEGs for different days.

### Construction of protein-protein interaction (PPI) network and screening of modules

We used the Search Tool for the Retrieval of Interacting Genes (STRING; version 11; string-db.org/) database to analyze the interactions among the DEGs. A cutoff value of greater than 0.4 was used to evaluate the protein-protein interaction (PPI) network. Visualization of the PPI networks was performed by Cytoscape (version 3.7.1, http://www.cytoscape.org/). The Cytoscape StringApp was used to retrieve the functional enrichment of up- and downregulated DEGs at days 8, 12, and 25. The top 10 proteins were ranked by topological analysis methods using CytoHubba. We found the most similar proteins in the following methods: MNC, degree, EPC, and closeness. Key modules in the PPI network were identified by using the Cytoscape plugin Molecular Complex Detection (MCODE, version 1.5.1). The criteria were both a node score and node number greater than 4.

### Cell culture

Human BM-MSCs were obtained from the Royan Stem Cell Bank. The study protocol was approved by the Royan Institute ethical committee board. All the experiments were designed in three biological replicates. The cells were cultured in α-MEM (Gibco, cat. no. 12571) with 10%FBS (Gibco, cat. no. 10082139), 100 U/ml penicillin (Gibco, cat. no. 15070063), and 100 mg/ml streptomycin. After 24 h, the cells were induced with osteogenic medium containing DMEM supplemented with 10%FBS, 50 μg/mL ascorbic acid (Sigma-Aldrich, cat. no. A8960), 10 mM β-glycerophosphate (Sigma-Aldrich, cat. no. 154804-51-0), and 1 × 10^−8^ M dexamethasone (Sigma-Aldrich, cat. no. D4902) for days 8, 12, and 25. The media was replaced every 3 days, and the characterization of osteogenic was performed by alizarin red and Oil Red O staining (Supplementary Fig 3).

### Validation of microarrays data by real-time PCR

The real-time PCR was performed for the validation of microarray data. The expression level of mRNAs for CTNNB1, MAPK3, ITGAV, ITGA5, CCNB1, and TLR4 were evaluated by a real-time PCR System. We isolated total RNA by TRI reagent (Sigma-Aldrich, T9424) from cultured MSCs in osteogenic medium on days 8, 12, and 25. cDNA was synthesized from 1 μg total RNA using the PrimeScriptTMRT reagent Kit (Takara Perfect Real Time). All reactions were carried out duplicate by using StepOnePlus Real-time PCR System (Applied biosystems life technologies, ABi). GAPDH was used as a reference gene to normalize the expression of all target genes. All tested groups compared to the control group (day 0). The △△Ct method was used to analyze Q-RT PCR data for quantitative analysis. The specific primers sequence for each gene is shown in Table 2.

**Table 2:** List of primers used for real-time PCR to validate microarray analysis

Target gene	Primer sequence	Accession number	Product size
GAPDH	F: CTCATTTCCTGGTATGACAACGA R: CTTCCTCTTGTGCTCTTG	NM_001357943.2	122 bp
CTNNB1	F: AATGCTTGGTTCACCAGTG R: GGCAGTCTGTCGTAATAGCC	NM_001330729.2	176 bp
MAPK3	F: TGACCATATCTGCTACTTCCTC R: GGTATAGCCCTTGGAGTTCAG	NM_001040056.3	250 bp
ITGAV	F: GCAACAGGCAATAGAGAT R: TGCTGAATCCTCCTTGACAA	NM_002210.5	262 bp
ITGA5	F: GCTGTGACTACTTTGCCGTG R: CGAGTTGTTGAGATTCTTGCTG	NM_002205.5	176 bp
CCNB1	F: GCTGGGTGTAGGTCCTTG R: CCTGCCATGTTGATCTTCG	NM_031966.4	149 bp
TLR4	F: TGATGTCTGCCTCGCGCCTG R: AACCACCTCCACGCAGGGCT	NM_138554.5	98 bp

### Statistical analysis

Statistical analyses were performed using Prism statistical software (version 8). The statistical significance of the differences between groups at days 8, 12, and 25 was performed by the Kruskal-Wallis test.

## Supplementary Information

**Additional file 1:.** Supplementary Figure 1. Venn diagram of molecular function (MF) and cellular component (CC) on days 8, 12, and 25. a Upregulated differentially expressed genes (DEG). b Downregulated DEGs. Supplementary Table 2. Hub genes for up- and downregulated genes ranked in CytoHubba. Supplementary Figure 2. Protein-protein interaction (PPI) network (STRING). Supplementary Figure 3. The ability of MSCs to differentiate into osteoblast and adipocyte

**Additional file 2:.** Supplementary Table 1. Biological process (BP) and KEGG enrichment analyses of the differentially expressed genes in the Modules. (XLS 42 kb)

## Data Availability

All data used and analyzed during this study are included in this published article and its supplementary information files. The other data including real-time PCR data are available from the corresponding author on reasonable request.

## Abbreviations

BM-MSC: Bone marrow-derived mesenchymal stem cell
DEG: Differentially expressed gene
GO: Gene ontology
LPS: Lipopolysaccharide
KEGG: Kyoto Encyclopedia of Genes and Genomes
MCODE: Molecular Complex Detection
PPI: Protein-protein interaction
ECM: Extracellular matrix
Runx2: Runt-related transcription factors 2
Dlx5: Distal-less homeobox 5
OCN: Osteocalcin
Osx: Osterix
BMP: Bone morphogenetic protein
GEO: Gene Expression Omnibus
BP: Biological processes
MF: Molecular function
CC: Cellular component
DAVID: Database for Annotation Visualization and Integrated Discovery
STRING: Search Tool for the Retrieval of Interacting Genes
ALP: Alkaline phosphatase
*VEGFB*: Vascular endothelial growth factor B
hADSC: Human adipose tissue-derived stem cell
hDPSC: Human dental pulp stem cell
PDGF: Platelet-derived growth factor
MAP2K2: Mitogen-activated protein kinase 2
PIK3R1: Phosphoinositide-3-kinase regulatory subunit 1
TSC: Tendon stem cell
MAPK: Mitogen-activated protein kinases
ERK1/2: Extracellular signal-regulated kinase-1/2
CDH11: Cadherin 11
ILK: Integrin-linked kinase
MKK: MAP kinase kinase
