# Supplementary material for: Microarray analysis identification of key pathways and interaction network of differential gene expressions during osteogenic differentiation
Source: Hum Genomics. 2020 Nov 25;14:43. doi: 10.1186/s40246-020-00293-1 (PMC7687700; doi:10.1186/s40246-020-00293-1)

**Supplementary information**

**Additional file 1: Supplementary Figure 1.** Venn diagram of molecular function (MF) and cellular component (CC) on days 8, 12, and 25. **a** Up-regulated differentially expressed genes (DEG). **b** Down-regulated DEGs.

­­
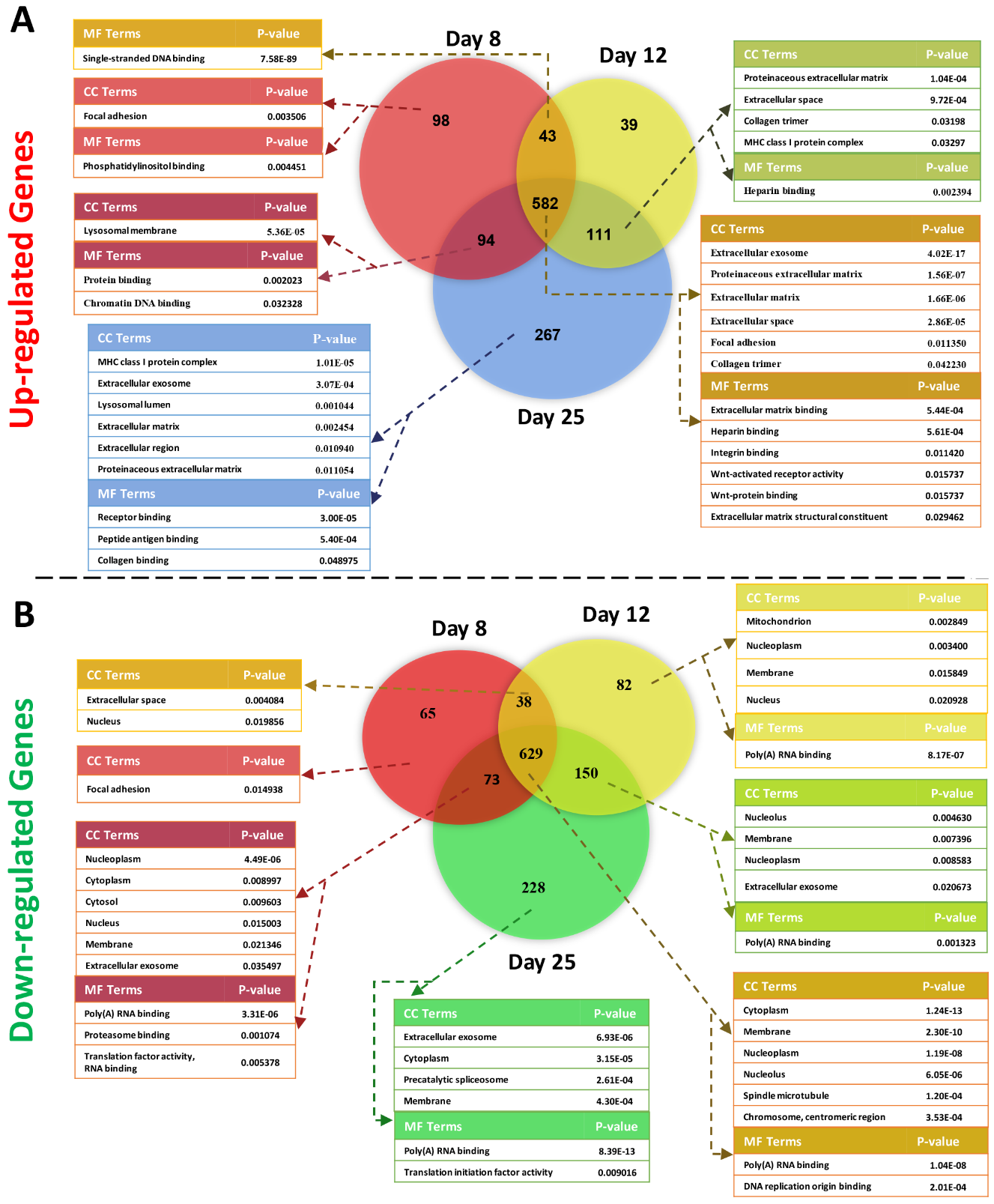


**Additional file 3: Supplementary Table 2.** Hub genes for up- and down-regulated genes ranked in CytoHubba

| **Day** | **Ranking Methods** | **Top-10-Nodes** |
| --- | --- | --- |
| **Day 8** | MNC | GAPDH-AKT1-CDK1-IL6-PLK1-VEGFA-CDC20-UBB-HSP90AA1-AURKA |
|  | Degree | GAPDH-AKT1-CDK1-IL6-PLK1-VEGFA-CDC20-HSP90AA1-AURKA-UBB |
|  | EPC | CDK1-PLK1-CDC20--AURKA-BIRC5-CCNA2-TOP2A-AURKB-MAD2L1-KIF11 |
|  | Closeness | GAPDH-AKT1-IL6-CDK1-VEGFA-HSP90AA1-MAPK3-JUN-UBB-CTNNB1 |
| **Day 12** | MNC | GAPDH-AKT1-CDK1-CCNB1-PLK1-CDC20-MAD2L1-IL6-AURKB-CCNA2 |
|  | Degree | GAPDH-AKT1-CDK1-CCNB1-PLK1-CDC20-MAD2L1-IL6-AURKB-AURKA |
|  | EPC | CDK1-CCNB1-CDC20-PLK1-AURKB-MAD2L1-AURKA-CCNA2-TOP2A-CENPF |
|  | Closeness | GAPDH-AKT1-CDK1-CCNB1-IL6-VEGFA-PLK1-HRAS-CCNA2-JUN |
| **Day 25** | MNC | GAPDH-AKT1-CDK1-IL6-PLK1-CCNB1-VEGFA-CDC20-HSP90AA1-AURKA |
|  | Degree | GAPDH-AKT1-CDK1-IL6-PLK1-CCNB1-VEGFA-CDC20-HSP90AA1-AURKA |
|  | EPC | CDK1-CTNNB1-PLK1-AURKA-CCNA2-CCNA2-AURKB-TOP2A-TOP2A-TPX2 |
|  | Closeness | GAPDH-AKT1-CDK1-IL6-VEGFA-HSP90AA1-HRAS-CCNB1-CTNNB1-PLK1 |

**Additional file 4: Supplementary Figure 2.** Protein-protein interaction (PPI) network (STRING). Presence of β-catenin in the protein-protein interaction (PPI) Network. Module 4 (day 25).

**_
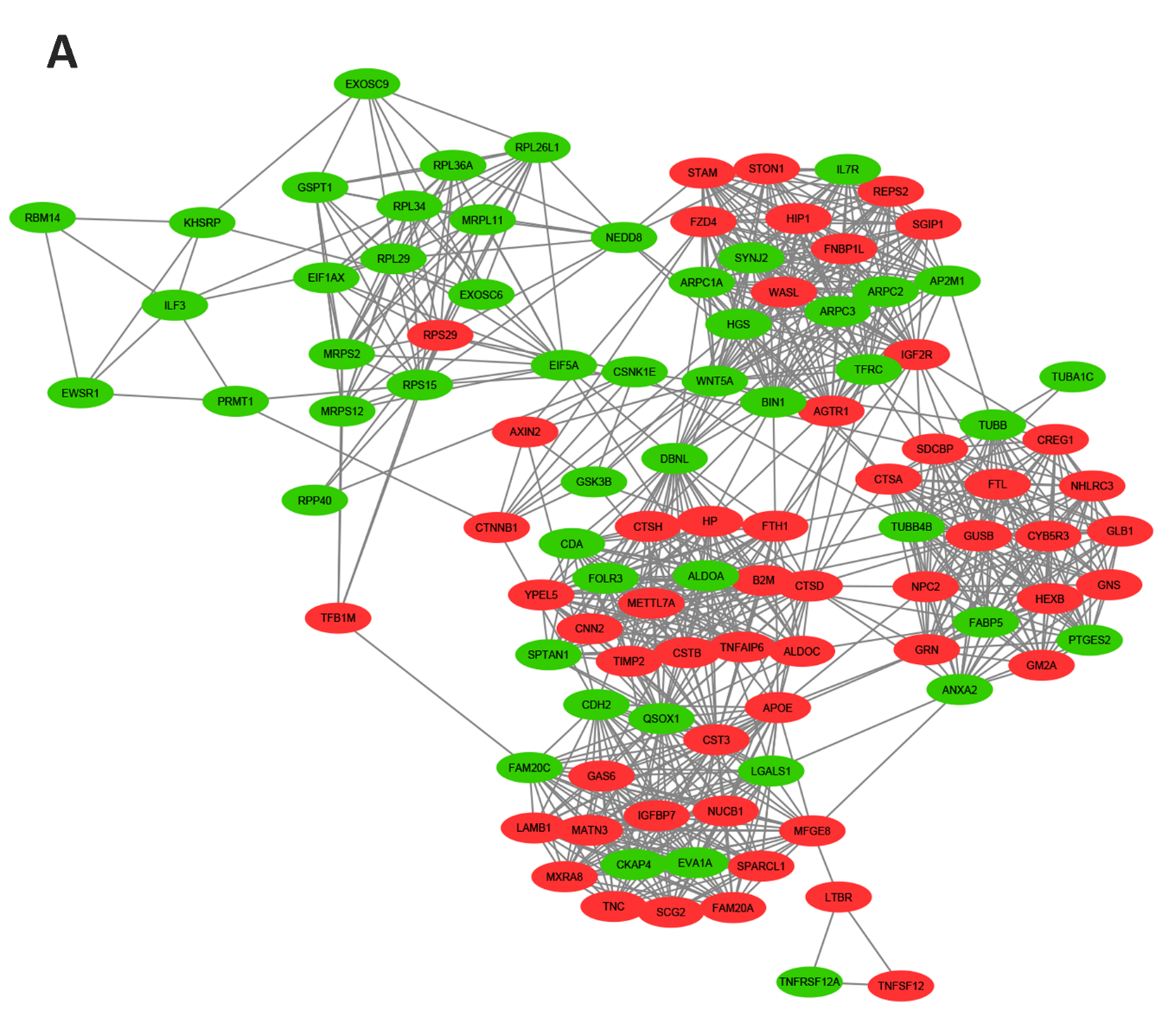
_**

**Additional file 5:** **Supplementary Figure 3.** The ability of MSCs to differentiate into osteoblast and adipocyte. MSCs were cultured in the osteogenic or adipogenic and basic medium for 25 days. Differentiation of MSC into osteoblasts and adipocytes were examined by alizarin red S and Oil Red O staining, respectively.


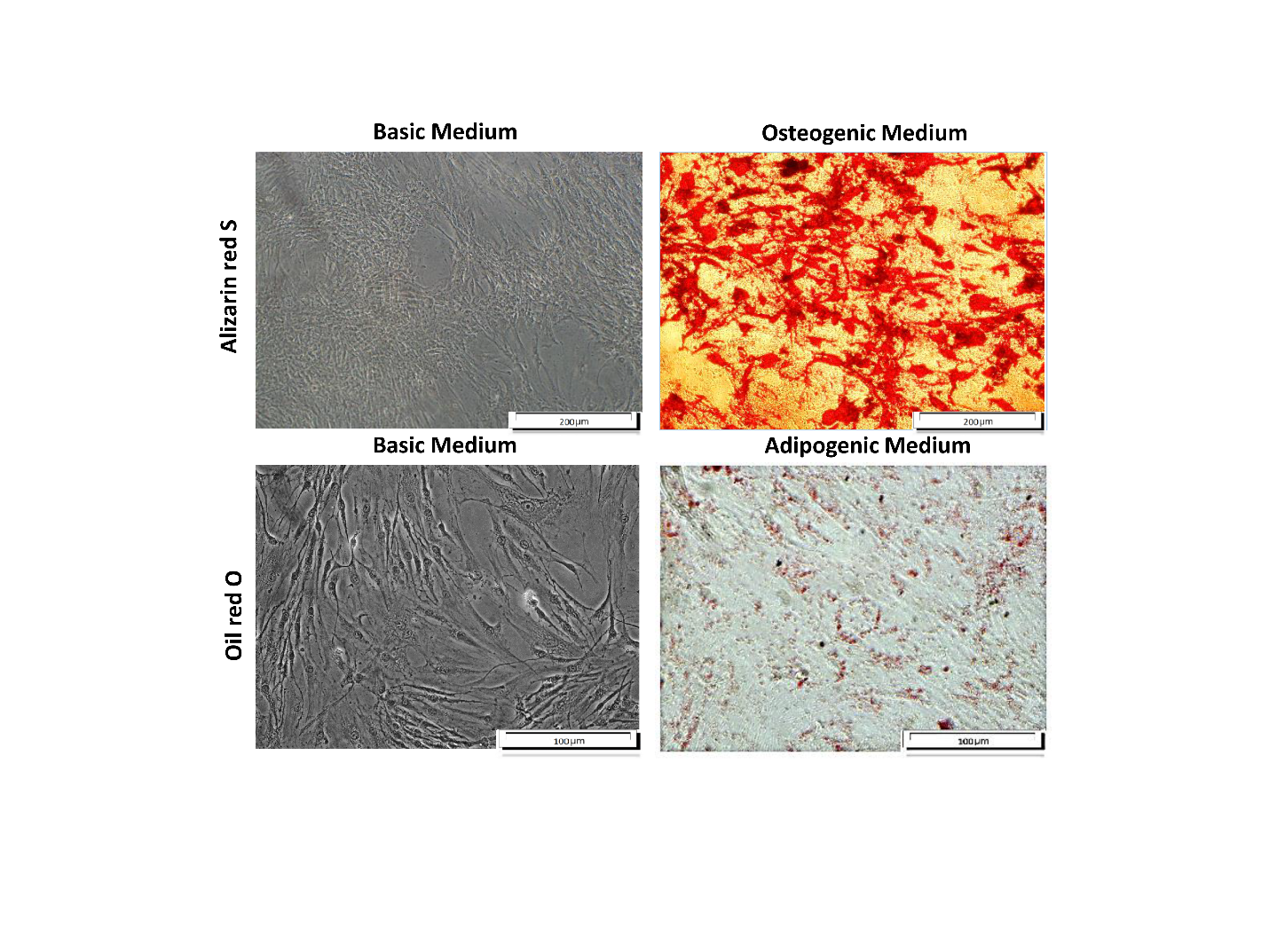

Supplement: Supplementary file 1 — Additional file 1:. Supplementary Figure 1. Venn diagram of molecular function (MF) and cellular component (CC) on days 8, 12, and 25. a Upregulated differentially expressed genes (DEG). b Downregulated DEGs. Supplementary Table 2. Hub genes for up- and downregulated genes ranked in CytoHubba. Supplementary Figure 2. Protein-protein interaction (PPI) network (STRING). Supplementary Figure 3. The ability of MSCs to differentiate into osteoblast and adipocyte [file 40246_2020_293_MOESM1_ESM.docx]
